# 8-Oxoadenine: A «New» Player of the Oxidative Stress in Mammals?

**DOI:** 10.3390/ijms25021342

**Published:** 2024-01-22

**Authors:** Alexander A. Kruchinin, Polina N. Kamzeeva, Dmitry O. Zharkov, Andrey V. Aralov, Alena V. Makarova

**Affiliations:** 1 Institute of Gene Biology, Russian Academy of Sciences, 34/5 Vavilova St., 119334 Moscow, Russia; kruchinin77@gmail.com (A.A.K.); polinabast@yandex.ru (P.N.K.); 2 National Research Center, Kurchatov Institute, Kurchatov sq. 2, 123182 Moscow, Russia; 3 Shemyakin-Ovchinnikov Institute of Bioorganic Chemistry, Russian Academy of Sciences, Miklukho-Maklaya 16/10, 117997 Moscow, Russia; baruh238@mail.ru; 4 Department of Natural Sciences, Novosibirsk State University, 1 Pirogova St., 630090 Novosibirsk, Russia; dzharkov@niboch.nsc.ru; 5 Institute of Chemical Biology and Fundamental Medicine, Siberian Branch of the Russian Academy of Sciences, 8 Lavrentieva Ave., 630090 Novosibirsk, Russia

**Keywords:** 7,8-dihydro-8-oxoadenine, mutagenesis, DNA polymerases, base excision repair, DNA glycosylases, translesion DNA synthesis

## Abstract

Numerous studies have shown that oxidative modifications of guanine (7,8-dihydro-8-oxoguanine, 8-oxoG) can affect cellular functions. 7,8-Dihydro-8-oxoadenine (8-oxoA) is another abundant paradigmatic ambiguous nucleobase but findings reported on the mutagenicity of 8-oxoA in bacterial and eukaryotic cells are incomplete and contradictory. Although several genotoxic studies have demonstrated the mutagenic potential of 8-oxoA in eukaryotic cells, very little biochemical and bioinformatics data about the mechanism of 8-oxoA-induced mutagenesis are available. In this review, we discuss dual coding properties of 8-oxoA, summarize historical and recent genotoxicity and biochemical studies, and address the main protective cellular mechanisms of response to 8-oxoA. We also discuss the available structural data for 8-oxoA bypass by different DNA polymerases as well as the mechanisms of 8-oxoA recognition by DNA repair enzymes.

## 1. Introduction

A number of environmental (ultraviolet and ionizing radiation, chemical oxidants [1]) and endogenous (products of oxidative phosphorylation in mitochondria [2] and metabolic oxidases [3]) factors induce the production of free radicals, particularly reactive oxygen species (ROS). These derivatives of molecular oxygen are involved in redox reactions and interact with biological macromolecules, causing their damage. DNA replication stress initiated by oxidative lesions is considered a hallmark of carcinogenesis. Furthermore, oxidative DNA damage contributes to aging and development of neurodegenerative, cardiovascular, and metabolic disorders [4,5].

ROS are a well-established cause of mutagenesis. Dozens of different nucleobase modifications resulting from oxidative stress have been found to arise in cells, whereas over a hundred distinct oxidative DNA lesions have been identified in vitro [6,7]. Guanine is regarded as the most vulnerable to ROS since it has the lowest redox potential among nucleobases [8]. The guanine C8 position is directly attacked by ROS, resulting in the production of 7,8-dihydro-8-oxoguanine (8-oxoG), the most abundant and comprehensively studied DNA lesion resulting from oxidative damage. This modified nucleobase can pair with both cytosine and adenine, leading in the latter case to G → T transversions upon replication. Adenine, which likewise has a relatively low redox potential, is also easily oxidized [8]. The data on the cellular levels of 7,8-dihydro-8-oxoadenine (8-oxoA) are inconsistent but generally vary from one-tenth to a half compared to that of 8-oxoG [9,10]. 8-oxoA also possesses ambiguous coding properties and is prone to oxidation itself. Nonetheless, 8-oxoA has received significantly less attention than 8-oxoG.

In this review, we focus on the miscoding potential and characteristics of 8-oxoA. We overview historical and recent genotoxicity studies of 8-oxoA and discuss its mutagenic potential in bacterial and eukaryotic cells. We also summarize the main cellular mechanisms of response to 8-oxoA and discuss the structural basis for 8-oxoA bypass by DNA polymerases as well as the mechanisms of 8-oxoA recognition by DNA repair enzymes.

## 2. Formation of 8-oxoA and Its Derivatives

**Figure 1:**
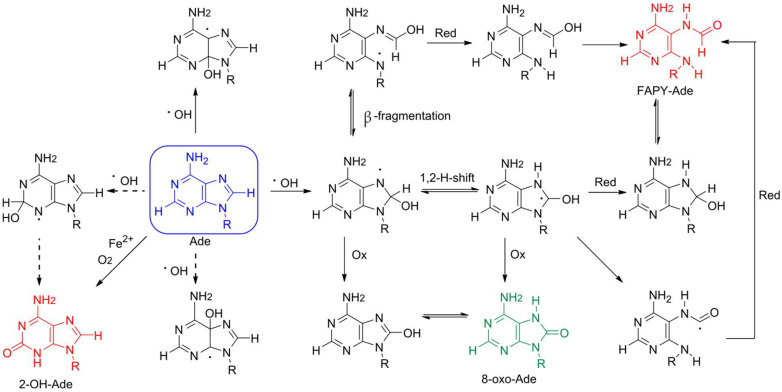
Scheme of putative pathways of adenine degradation. The parent adenine and products of its oxidation are highlighted in blue (adenine), green (7,8-dihydro-8-oxoadenine), and red (2-hydroxyadenine and 4,6-diamino-5-formamidopyrimidine). Ox and red stand for oxidation and reduction, respectively. The ineffective processes are shown with dashed arrows.

Adenine is oxidized less efficiently due to its higher redox potential compared with guanine [8,11]. The oxidation of adenine to 8-oxoA upon γ-radiation was discovered in aqueous solution [12,13] and then found in DNA samples [14,15], murine chromatin [7], as well as in DNA of tumor cells [16]. This lesion is predominantly formed through the exposure of hydroxyl radical on adenine [13], which is generated by water ionization upon γ-radiation or as the result of peroxide degradation through the Fenton reaction [1,13], whereas singlet oxygen does not contribute to 8-oxoA formation (Figure 1) [1]. Based on studies of dA, dATP, and DNA oxidation using the Fe^2+^/EDTA system with a high Fe^2+^ concentration, 2-hydroxy-2′-deoxyadenosine (2-OH-dA) was previously considered as the main lesion resulting from 2′-deoxyadenosine damage [17,18]. Thus, extensive studies on 2-OH-dA incorporation into DNA, nucleotide incorporation opposite 2-OH-dA and its repair pathways were performed [19,20,21,22]. However, in contrast to non-physiological conditions with high Fe^2+^ concentration and, presumably, iron autoxidation [18], high performance liquid chromatography (HPLC) separation associated with tandem mass spectrometry (MS/MS) detection revealed 8-oxoA is a primary stable product both upon γ-irradiation and under Fenton reaction conditions [18,23,24]. Along with 8-oxoA, the formation of 4,6-diamino-5-formamidopyrimidine (FapyA) also occurs as a result of attack by hydroxyl radicals (Figure 1), but it tends to isomerize and become hydrolyzed in aqueous conditions [23].

**Figure 2:**
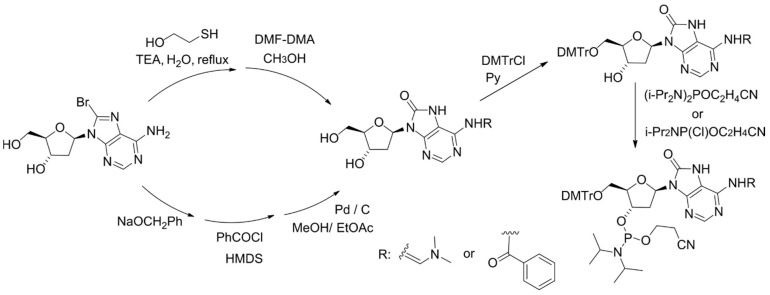
Scheme for the synthesis of 8-oxoA phosphoramidite with a key step using sodium benzylate or 2-mercaptoethanol.

For physicochemical and biological studies of 8-oxoA-containing DNA fragments, the method for chemical introduction of 8-oxoA 2′-deoxynucleotide into oligonucleotides has been developed. The key step of the synthesis is based on the conversion of 8-bromo-2′-deoxyadenosine (8-Br-dA) into a 8-benzyloxy-substituted derivative, followed by its catalytic hydrogenation [25], or on treatment of 8-Br-dA with a mixture of 2-mercaptoethanol and triethylamine (Figure 2) [26,27].

## 3. Mutagenic Potential of 8-oxoA

### 3.1. Pro-Mutagenic Nature of 8-oxoA

Some oxidative DNA modifications are cytotoxic. For example, enantiomers of thymine glycol significantly distort the geometry of the DNA molecule and represent a serious obstacle for high-fidelity replicative DNA polymerases [28]. The accumulation of distorting adducts may be accompanied by replication arrest, chromosome aberrations, and activation of apoptosis. Nevertheless, most non-bulky oxidative DNA lesions are miscoding rather than blocking. 8-oxoG is known to induce G → T transversions in DNA due to its pairing with dA, when the modified base is in the *syn* orientation. 8-oxoG can be ambiguously read by replicative enzymes leading to mutations after the next round of replication. In turn, the available evidence suggests that 8-oxoA in eukaryotic cells has a moderate mutagenic potential rather than showing blocking effects (see Section 3.3).

**Figure 3:**
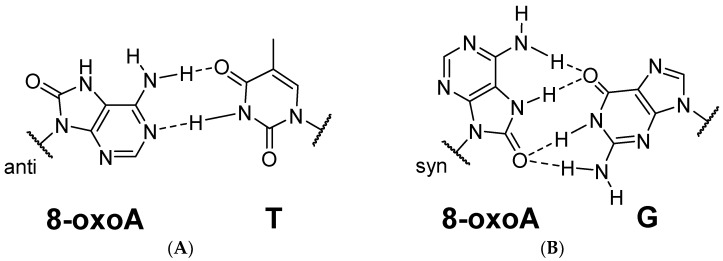
Pairing of 8-oxoA with thymine (**A**) or guanine (**B**).

Mutagenic potential of 8-oxoA, like that of 8-oxoG, is determined by its ability for ambiguous base pairing. Mutagenicity of DNA lesions is usually considered to depend on the stability of the potential mismatches [29]. The Watson–Crick face of 8-oxoA remains intact allowing the formation of a stable base pair with thymine (Figure 3A). According to NMR and thermal melting studies, the 8-oxoA:T pair does not substantially disrupt the helix geometry [30] but slightly decreases T_m_ (by 1.7 °C in a 30-bp duplex) [31]. Some moderate distinctions in the backbone structure of the oligonucleotide containing 8-oxoA:T pair, compare to the A:T pair, were captured with Fourier transform-infrared spectroscopy [32]. In addition, fine structure analysis of the DNA duplex with 8-oxoA:C pair, in contrast to A:T, 8-oxoA:T, and A:C pairs, demonstrated notable structural and dynamic differences [31]. The spatial structure of the damaged region can also influence the recognition by DNA metabolism enzymes [29]. Indeed, the presence of few mismatches with 8-oxoA impedes the recognition of the damaged site by DNA glycosylases (see Section 4) [31]. It is likely that the mild distortion of the conformational geometry of the sugar-phosphate backbone caused by 8-oxoA in DNA might in some degree inhibit the activity of at least high-fidelity DNA polymerases.

Of particular interest is the ability of 8-oxoA to pair with guanine. According to X-ray studies on the 8-oxoA:G pair, the modified base also adopts the *syn* conformation, wherein its position remains unchanged compared to that in the A:G pair, while G is shifted into the minor groove. The 8-oxoA:G pair was proposed to be held together by two three-centered hydrogen bonds (Figure 3B) [33]. Interestingly, the angles between the glycosidic bonds and the C1′-C1′ distance in the pair are almost equal, which is characteristic of Watson–Crick pairs, while asymmetry in these angles is typically observed in *syn-anti* purine–purine pairs [33]. The formation of the 8-oxoA:G pair could explain the observed A → C mutations. DNA duplex containing two 8-oxoA:G pairs also adopts the standard B-form without significant geometry distortions [33]. Overall, the discriminating ability of 8-oxoA for the opposite base is lower than that of the parent adenine [31]. The modification was shown to diminish mismatch discrimination with the highest T_m_ of 54.7 °C and 51.2 °C for 8-oxoA:T and 8-oxoA:G pairs, respectively, in a 13-bp DNA duplex [27].

Both 8-oxoG and 8-oxoA exist in tautomeric equilibrium, with the O^8^ keto form greatly predominating over the enolate form in an aqueous solution. Biological implication of tautomerism is essential for understanding base pairing [34]. According to X-ray and NMR studies, 8-oxoA paired with G and T exists predominantly in the keto form in DNA duplex [30,33,35], while its enolate form is proposed for an 8-oxoA:G base pair in the catalytic site of human DNA polymerases Pol β and Pol η [36]. Although the genotoxicity of the lesion is largely determined by its ability to form non-complementary base pairs, the configuration and stability of the base pair in oligonucleotides and in the catalytic center of an enzyme may differ due to the involvement of the amino acid microenvironment in the pair stabilization.

It was suggested that base pairs containing 8-oxoA appear to be more susceptible to the microenvironment (e.g., pH and Me^2+^ ions affecting ionization and tautomerization) compared to 8-oxoG [36]. In particular, 8-oxoG:dATP mismatch adopts the same conformations both in duplex DNA and in the active sites regardless of the enzyme [37,38,39,40], while the available conformations of 8-oxoA:dGTP mismatch are different, highlighting the greater impact of the microenvironment on the stability of 8-oxoA base pairs [33,36]. A comparative mutagenesis assay in mammalian cells also demonstrated the impact of DNA sequence context on the mutagenic potential of 8-oxoA (see Section 3.3 and Section 7) [37].

### 3.2. 8-oxoA-Induced Mutagenesis in Prokaryotes

Different studies have yielded conflicting estimates of the mutagenic potential of 8-oxoA in vivo. Based on a limited number of studies, 8-oxoA does not appear to pose a serious threat in *Escherichia coli*. In contrast to 8-oxoG, mutation analysis in bacterial cells transformed with a single-stranded DNA substrate containing 8-oxoA demonstrated very low mutagenic potential [41] (Table 1). These results are in good agreement with in vitro studies, which show that Klenow fragments (KF) of Pol I and Taq DNA polymerase bypass 8-oxoA in an error-free manner, almost independently of DNA sequence context [30,42,43,44] (Table 1). A very small number of substitutions associated with the insertion of non-complementary dAMP and dGMP opposite 8-oxoA by KF was reported only in one study [45]. In the presence of all four dNTPs, primer extension by KF was moderately inhibited; however, a 10-fold excess of the enzyme overcame this suppression. These observations suggest that the miscoding potential of 8-oxoA in bacterial cells is highly limited.

### 3.3. 8-oxoA-Induced Mutagenesis in Mammalian Cells

Initial data from HeLa and COS-7 cell extracts revealed rather negligible mutagenic properties of 8-oxoA (Table 2) [48]. However, the presence of 8-oxoA in mouse embryonic fibroblasts led to A:T → C:G transversions and A:T → G:C transitions at a relatively high frequency comparable to that caused by 8-oxoG [42,49]. In this work, Kamiya et al. used a DNA substrate representing a fragment of the *HRAS* oncogene with the 8-oxoA lesion at a mutational hotspot [42]. Another comparative mutagenesis study by Tan et al. found that the mutagenic potential of 8-oxoA placed in the non-coding strand of the *HRAS* gene is approximately four-fold lower than that of 8-oxoG (1.2% vs. 5.2%). In contrast to 8-oxoG, the oxidized adenine placed in a different position did not show any significant mutagenic properties [50].

As reviewed in [51], high-fidelity eukaryotic DNA polymerases α, δ, and ε preferentially incorporate non-complementary dAMP opposite 8-oxoG in vitro resulting in G:C → T:A transversions. In contrast to guanine modification, the activity of eukaryotic DNA polymerases opposite 8-oxoA remains poorly characterized, even for high-fidelity replicative DNA polymerases, with the exception of Pol α. In the presence of each individual dNTP, calf thymus Pol α performs accurate synthesis past 8-oxoA; however, the polymerase activity of the enzyme is moderately inhibited [43]. Another study showed that the murine DNA polymerase α-primase complex not only inserts the correct nucleotide opposite the lesion but also incorporates a small amount of dGMP when 8-oxoA is placed in the *HRAS* oncogene fragment as a template [42], in line with the in vivo *HRAS* mutagenesis studies.

Therefore, the mutagenic potential of 8-oxoA is prominent in mammalian cells where it appears to depend on the DNA sequence context, but is greatly minimized in bacteria. This phenomenon can be driven by differences in the accuracy of high-fidelity and TLS polymerases opposite 8-oxoA or differences in base excision repair (BER) in prokaryotic and eukaryotic cells.

### 3.4. Incorporation of 8-oxodATP

Oxidation of purine nucleobases also takes place in the nucleotide pool. 8-oxodGTP can be incorporated into the genome with relatively high efficiency [52,53]. MTH1 hydrolyzes 8-oxodGTP in the nucleotide pool and thereby prevents the mutagenic incorporation of oxidized dGTP into DNA [54]. It was also demonstrated that MTH1 has similar activity towards 8-oxodATP [22]; however, the discrimination ability of DNA polymerases regarding oxidized dATP remains poorly understood. Limited studies in vitro indicate that 8-oxodATP is probably an inferior substrate for DNA polymerases, but its mutagenic effect in vivo cannot be completely ruled out. Pol β demonstrated the most efficient incorporation of 8-oxodATP, especially opposite template T [55]. KF and Pol α catalyzed the reaction with a relatively low efficiency, preferentially opposite non-complementary template G and A, while Pol λ was unable to incorporate 8-oxodATP on any DNA substrate [55,56].

## 4. The Effects of 8-oxoA Persistence in Genome

Depending on the detection technique, analyzed tissue samples or body fluids, and a number of other factors, the amount of oxidized adenine and guanine modifications in DNA can vary significantly [57]. The cellular background level of 8-oxoG is in the range of 1000 to 2000 lesions, formed daily in a single human cell under physiological conditions [58,59,60,61]. In early works, the number of 8-oxoA lesions ranged from 10 to 50% of 8-oxoG, while one later study detected 8-oxoA at 0.7 lesions per 10^6^ nucleotides, which corresponds to ~2200 lesions per human genome and is comparable with 8-oxoG levels [14,62]. The amount of 8-oxoA induced by ionizing radiation or hydrogen peroxide is about 2–3 times lower than that of 8-oxoG [14,63]. Elevated levels of both adenine and guanine oxidized modifications have been found in many mammalian tumor tissues (stomach, larynx, ovary, brain, and lung) [10,16] and the ratio of 8-oxoA to 8-oxoG reaches 1:1 in some of them [10]. The endogenous levels of both 8-oxoG and 8-oxoA are doubled in aged rat tissues compared to young tissues [64]. Thus, the abundance of 8-oxoA in genome is on a par with 8-oxoG.

Oxidative stress-induced mutagenesis is a crucial component of cancer pathogenesis. Both 8-oxoA and 8-oxoG induce mutations in the synthetic *HRAS* proto-oncogene, resulting in its activation and suggesting the role in carcinogenesis [42,50,65]. The ROS-induced C → A mutations (or G → T in the complementary strand) corresponding to cancer signatures SBS18 and SBS36 have been attributed to 8-oxoG and described in human tumors (primarily in colorectal and pancreatic cancer for SBS36 and in neuroblastoma, gastrointestinal cancer, and breast cancer for SBS18) [66,67].

**Figure 4:**
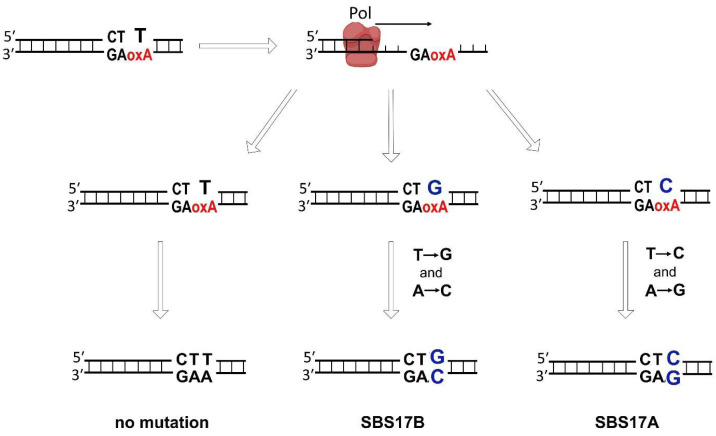
Proposed alternative mechanism of 8-oxoA-induced mutagenesis for cancer signatures SBS17a and SBS17b.

8-oxoA-specific substitutions T → G and T → C most frequently observed in the CTT context (or A → C and A → G in the AAG context similar with the *HRAS* CC**XAG** mutagenesis hot spot) have been also detected in esophageal and stomach adenocarcinoma, B-cell non-Hodgkin lymphoma, and pancreatic cancer, and correspond to signatures SBS17a (T → C) and SBS17b (T → G) [https://cancer.sanger.ac.uk/signatures/sbs/sbs17b/ (accessed on 15 February 2023)] [68,69]. These signatures have strong correlation with replication timing, lagging-strand enrichment and are linked with DNA damage [69]. The mutational process causing Signatures 17 remains unclear; some cases of SBS17b are associated with 5-fluorouracil therapy [70] but in its absence could be connected to the incorporation of oxidized dGTP opposite template A by TLS polymerases [69]. We suggest that 8-oxoA-induced mutagenesis can be an alternative driver for Signature 17-associated mutagenesis (Figure 4). Since duodeno-gastric reflux causes oxidative stress, this hypothesis also explains the prevalence of these mutational patterns in gastric and esophageal cancers.

Defects in OGG1, MUTYH, MTH1, NEIL1, and APE1 associated with 8-oxoG repair, often lead to the accumulation of DNA single-strand breaks (SSB) and other intermediates of incomplete repair [71,72,73,74,75,76]. Knockout mice with impaired OGG1, MUTYH, or MTH1 genes had increased frequency of 8-oxoG-induced G → T mutations in the adenomatous polyposis coli (*APC*) tumor suppressor gene and *KRAS* proto-oncogene resulting in a dramatic increase in the incidence of tumors [72,74]. However, 8-oxoA-specific T → G and T → C mutations are yet to be reported in BER-knockout models.

It is hard to overestimate the clinical relevance of oxidative stress. 8-oxoG- and 8-oxoA-associated mutagenesis is not limited by the increased risk of mutations in cancer driver genes. The persistence of 8-oxoG is considered to be an important element in the pathogenesis of neurodegenerative diseases such as Alzheimer’s disease [77] and Parkinson’s disease [78]. The accumulation of 8-oxoG and 8-oxoA was found in patients with Cockayne syndrome, a genetic human disease with clinical symptoms that include neurodegeneration and premature aging (see also Section 5) [62,79].

**Figure 5:**
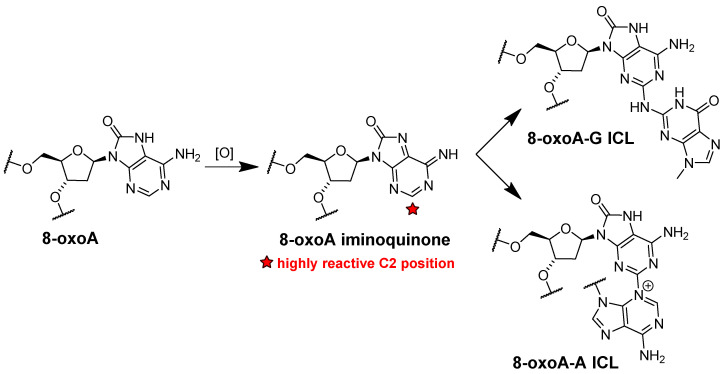
Proposed pathways for ICLs formation between the product of the further 8-oxoA oxidation and guanine or adenine in the opposite strand.

The genotoxic effect of both 8-oxoG and 8-oxoA is not limited to its mutagenic potential. It was demonstrated that 8-oxoA facilitates formation of other DNA lesions. Since the redox potential of 8-oxoA is significantly lower than that of adenine, further oxidation of the modified base could occur [80]. The highly reactive 8-oxoA iminoquinone could react easily at the C2 position with nucleophilic reagents [80]. Indeed, during further oxidation of 8-oxoA in DNA, interstrand cross-links (ICLs) arise with a nucleobase flanking the nucleotide opposite to the lesion. The most efficiently formed ICLs with adenine and guanine are shown in Figure 5 [80].

DNA lesions induced by oxidative stress can form oxidative clustered DNA lesions or multiply damaged sites [81,82]. 8-oxoA, together with 8-oxoG, 5-hydroxycytosine and apurinic/apyrimidinic (AP) sites, was shown to occur in clusters of lesions produced in DNA by ionizing radiation [83]. Such damage clusters may significantly distort DNA structure and present serious obstacles to both replication fork and BER enzymes, resulting in double-strand break (DSB) formation and modulating mutagenic potential of 8-oxoG [31,84,85,86]. It was also shown that 8-oxoA could suppress the repair of neighboring AP sites, 8-oxoG and oxidized pyrimidines [87,88,89]. On the other hand, human thymine–DNA glycosylase (TDG) excises normal T placed opposite to 8-oxoA, providing an opportunity for double-strand break formation (see below) and potentially mutagenic DNA repair synthesis [90].

Both 8-oxoG and 8-oxoA are also capable of inhibiting the exonuclease activity of the WRN helicase required to restart replication after fork arrest in vitro [91] and stimulated by Ku70/Ku80 (XRCC6/XRCC5), a double-strand break sensor, to digest oxidized DNA strands [91,92]. In addition, this helicase may be involved in the suppression of 8-oxoG-induced mutagenesis [93]. Finally, 8-oxoA has been reported to affect the activity of topoisomerases, although the magnitude of the effect is only severalfold and its direction seems to depend on the enzyme; while the cleavage by human topoisomerase II is accelerated [94], the vaccinia virus enzyme (type I) is inhibited by 8-oxoA [95].

Moreover, it is possible that persistence of 8-oxoA in genomic DNA can affect transcription efficiency and accuracy. The presence of 8-oxoG and its repair intermediates, such as AP sites, in the promoter region inhibits gene transcription by the impeding of transcription factor binding [96,97,98,99]. On the other hand, accumulation of 8-oxoG activates the Ras-MEK-MyoD signal axis enhancing the expression of regulatory factors and the differentiation of muscle tissue cells [100]. Oxidative DNA lesions may lead to so-called transcriptional mutations. In particular, 8-oxoG in the coding DNA strand can be incorrectly transcribed by RNA polymerase II, avoiding its fidelity checkpoints and thereby leading to C → A transversions in mRNA [101,102]. Since the presence of the DNA lesions correlates with functional impairments in non-proliferating neurons, the phenomenon of transcriptional mutation has been proposed as a possible mechanism for the formation of neurotoxic proteins that may be a potential cause of alpha synuclein aggregation in Parkinson’s disease [78]. In addition, transcriptional mutations induced by 8-oxoG greatly affect in vivo splicing fidelity [103]. The effect of 8-oxoA on transcription is yet to be characterized. However, 8-oxoA has been already shown to inhibit the activity of RNA polymerase II in vitro [104].

## 5. Repair of 8-oxoA

To mitigate the harmful effects of oxidative stress-induced DNA lesions, cells possess a collection of repair systems. Similar to its guanine counterpart, most of 8-oxoA must be removed before the next round of replication. The repair of 8-oxoA seems to be an efficient process since the half-life of H_2_O_2_-induced 8-oxoA in human cells is 4.6-fold shorter than the half-life of 8-oxoG lesion [63].

As a close chemical analog of 8-oxoG, 8-oxoA is generally believed to be repaired through the BER pathway. However, a DNA glycosylase that would remove 8-oxoA as its primary, or even a major substrate, has long been elusive (Table 3). Fpg, the main bacterial DNA glycosylase for 8-oxoG, excises 8-oxoA from 8-oxoA:T pairs in oligonucleotide substrates with a ~2300-fold lower specificity constant (*k*_cat_/*K*_M_) than 8-oxoG paired with C and 68,000-fold worse than 8-oxoG paired with T [105]. Removal of 8-oxoA paired with C by Fpg was reported at the level of <1% of 8-oxoG:C [106]. In another assay that uses γ-irradiated calf thymus DNA (which contains only 8-oxoA:T) and measures the release of the free base by chromatography/mass spectrometry, 8-oxoA release was found to be at least tenfold less efficient than the excision of 8-oxoG [107,108,109]. The structure of Fpg reveals a tight network of hydrogen bonds donated to O^6^ of the damaged base everted into the enzyme’s active site [110,111], which would obviously be disrupted if an exocyclic amino group is present instead, as in 8-oxoA. Molecular dynamic modeling indicates that 8-oxoA forms notably fewer bonds than 8-oxoG in the active site of Fpg [112]. Finally, *E. coli* MutY, an enzyme that processes A:8-oxoG and A:G mispairs, tightly binds 8-oxoA:8-oxoG and 8-oxoA:G but does not excise 8-oxoA from them [113]. Together, these observations suggest that the bacterial system of 8-oxoG repair is probably not involved in the removal of 8-oxoA.

**Figure 6:**
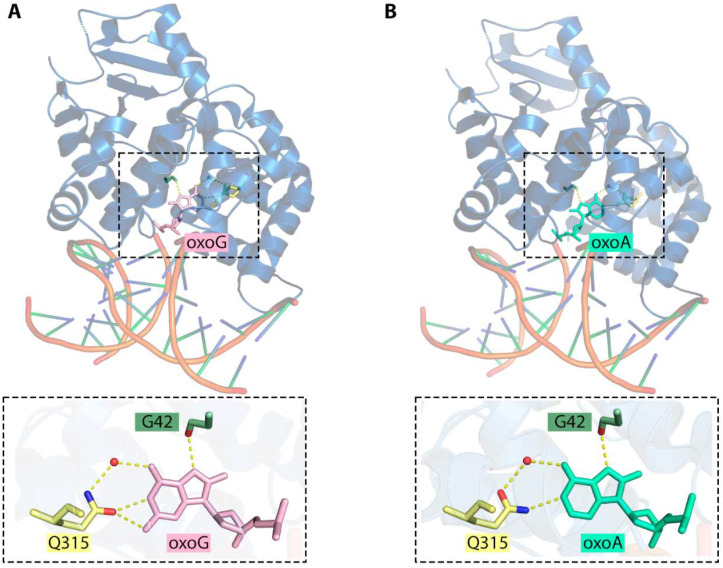
(**A**) Interactions in the active site of OGG1 bound to 8-oxoG (PDB ID: 1EBM) [123]. (**B**) The proposed model of 8-oxoA binding based on the OGG1:8-oxoG structure (PDB ID: 1EBM). Hydrogen bonds between nucleobases and interactions with the enzyme amino acids are represented with dashed lines; water molecules are indicated as red spheres. The Q315 and G42 O atoms are shown in red and N in blue.

In mammalian cells, 8-oxoG is predominantly removed by OGG1 DNA glycosylase whereas NEIL1, NEIL2, and NEIL3 may excise other products of oxidative damage to purines [114,115]. When assayed on duplex oligonucleotides, human, mouse and yeast OGG1 do not excise 8-oxoA from pairs with T or they do it with a much lower efficiency in comparison with their natural 8-oxoG substrate [106,116,117]. The same is true for the irradiated DNA assay: the base is specifically missing from the products of treatment of damaged calf thymus DNA by human, *Drosophila*, *Arabidopsis,* and yeast OGG1 [118,119,120,121]. Interestingly, however, OGG1 excises 8-oxoA even more efficiently than 8-oxoG when 8-oxoA is mispaired with C [106,117,122]. This would require a 180° rotation of the Gln315 side chain, which normally forms a bifurcated hydrogen bond to N1 and N^2^ of 8-oxoG in the enzyme’s active site through its Oε1 atom [123]. Flipping the side chain amide would allow Gln315 to reverse the hydrogen bond donor and acceptor and maintain the bond with N1 (Figure 6). After the damaged base is removed, APE1, the major AP endonuclease in human cells, stimulates OGG1 by displacing it from the enzyme–product complex and enhancing the turnover [124,125,126]. Again, this stimulation is robustly observed with 8-oxoA:C substrate but not with 8-oxoA:T [127].

NEIL1, which displays a rather mediocre activity on 8-oxoG, efficiently excises 8-oxoA from 8-oxoA:C and channels BER into the APE1-independent, polynucleotide kinase/3′-phosphatase dependent branch [128]. Also, an unidentified activity in human mitochondria was reported to excise 8-oxoA paired with G [122]. Overall, it seems that at least the repair of 8-oxoA:C in eukaryotes might be protective, which would only make sense if incorporation of 8-oxoA from the oxidized dNTP pool occurs. It remains to be seen whether this is the case and which polymerase may be responsible. However, 8-oxodATP is an excellent substrate for human MTH1, the dNTP pool sanitizing enzyme, which indirectly supports the possibility of its detrimental effects through misincorporation [22].

Recent reports highlighted a possible role of two members of the α/β-fold uracil DNA glycosylase superfamily, human TDG and *E. coli* Mug, in the repair of 8-oxoA [129,130]. Despite some disagreement on the activity of Mug and the order of preference for different mispairs, both studies detected the removal of 8-oxoA by TDG opposite from any base with efficiency comparable with or better than that for the established substrates such as 3,*N*^4^-ethenocytosine, T:G, U:G and oxidized/deaminated 5-methylcytosine derivatives. The biological significance of these observations is presently unclear. Since TDG can also remove T paired with 8-oxoA and AP sites [90] the co-existence of these activities can lead to clustered AP sites or double-strand DNA breaks. Moreover, Tdg-null mouse embryonic fibroblasts are not hypersensitive to ionizing radiation [129]. Thus, while TDG (and possibly Mug) can be considered as candidates for 8-oxoA repair in its natural 8-oxoA:T context, more data are required to validate this role.

Evidence for the involvement of other DNA repair pathways in the removal of 8-oxoA is very limited. Cockayne syndrome B (CSB, also known as ERCC6) protein is apparently involved, since cells from Cockayne syndrome B patients are deficient in their ability to clear 8-oxoA [62,79]. The mechanism is presently unclear although it might be related to stimulation of OGG1, which is known to participate in a multiprotein complex involving CSB [131,132]. Moreover, the rate of 8-oxoA removal is decreased in *XPC*-deficient cells, pointing to possible involvement of global genomic nucleotide excision repair [133].

**Table 3:** DNA glycosylases that may be involved in BER of 8-oxoA. The excised base is bold.

Organism	DNA Glycosylase	Base Pair	Reference
Prokaryotes	MUG	**8A**:T	[129]
		**8A**:G	
		**8A**:C	
		**8A**:A (low activity)	[130]
		**8A**:C (low activity)	
	Fpg	**8A**:C (low activity)	[106,107,108,109]
		γ-irradiated DNA (low activity)	
Eukaryotes	OGG1	**8A**:C	[106,117,122,127]
		**8A**:5-mC	
	TDG	**8A**:T	[90,129]
		**8A**:G	
		**8A**:C	
		**T**:8A	
		**8A**:G (+++)	[130]
		**8A**:C	
		**8A**:A	
		**8A**:T	
	NEIL1	**8A**:C	[128]
	Unidentified mitochondrial enzyme, distinct from OGG1	**8A**:G	[122]

+++—high activity.

## 6. Translesion DNA Synthesis Opposite 8-oxoA: The Second Chance to Avoid Harmful Effects

Although BER is a highly efficient mechanism for removing oxidized modifications from the genome, some of the damaged nucleotides are able to escape from the repair machinery. The concept of TLS implies that specialized DNA polymerases possessing a unique active site are able to bypass various lesions, including 8-oxoG, with high efficiency and accuracy. However, is this true for 8-oxoA? To date, the TLS activity opposite 8-oxoA has been characterized biochemically and structurally only for three DNA polymerases involved in TLS and repair: human Pol β from X family, human Pol η, and archaeal Dpo4 from Y family [5,36,46,47].

### 6.1. 8-oxoA Bypass by DNA Polymerase β

Pol β, belonging to the X family of DNA polymerases and playing a key role in BER, is best characterized both biochemically and structurally due to its monomeric nature, small size, and simplicity of purification.

Although Pol β demonstrates a moderate (more than twofold) decrease in the efficiency of nucleotide incorporation opposite 8-oxoA on a single-nucleotide gapped substrate, the lesion does not seem to represent a serious obstacle for the enzyme [36]. However, Pol β inserts non-complementary dGTP opposite 8-oxoA with a higher efficiency than opposite undamaged adenine supporting the pro-mutagenic properties of the lesion. The relative efficiency of dGTP insertion opposite 8-oxoA (8.3 × 10^−2^) is more than 400-fold higher than opposite intact nucleobase (1.9 × 10^−4^) (Table 2) [36]. Compared to the extension of undamaged DNA, the elongation of DNA strand from 8-oxoA:dTTP and 8-oxoA:dGTP pairs by Pol β is carried out with a ~40-fold decreased *k*_cat_/*K*_m_. The extension from 8-oxoA:dTTP pair is ~4-fold more efficient than from 8-oxoA:dGTP (Table 2).

The catalytic activity and fidelity of DNA polymerases may be significantly affected by microenvironmental components such as pH, metal ions, and base modifications. Upon the change in pH, as well as the formation of minor groove interactions induced by the modification of the nucleobase, ionization or tautomerization can take place, leading to the base transition from the keto form to the enolate intermediate and enol tautomer, which differ in base pairing properties. Indeed, an elevated pH level in the reaction buffer significantly facilitates the mutagenic incorporation of dGMP opposite both undamaged A (10-fold) and 8-oxoA (7-fold) by Pol β [36] and possibly by other polymerases.

Pol β, like high-fidelity DNA polymerases, undergoes sequential reconfiguration of the active site during catalysis between two states: an inactive open state and a catalytically active closed one. Large-scale conformational shifts of the Pol β active site are dependent on the incoming nucleotide and are triggered by its binding. The catalytically competent state of the active site is achieved by a closing movement of the thumb subdomain and rotation of α-N helix of the fingers subdomain.

**Figure 7:**
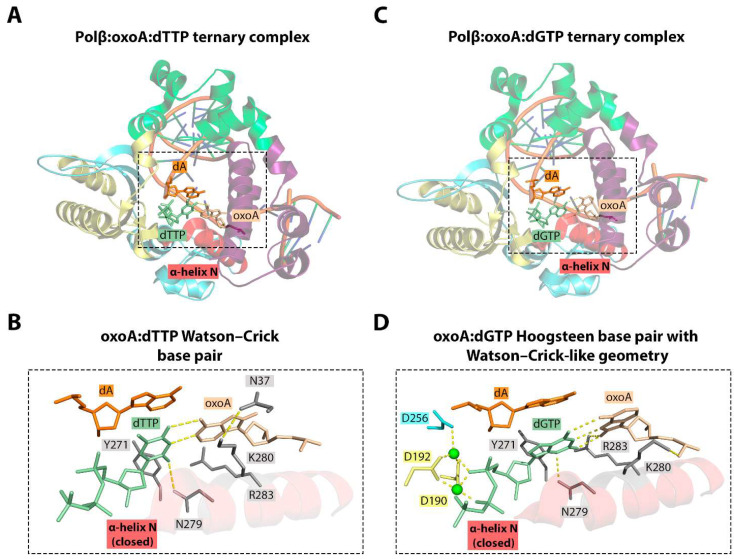
(**A**) Structure of the Polβ:oxoA:dTTP ternary complex (PDB ID: 6E3V) [36]. (**B**) Close-up view of the active site of the Polβ:oxoA:dTTP complex. (**C**) Structure of the Polβ:oxoA:dGTP ternary complex (PDB ID: 6E3W) [36]. (**D**) Close-up view of the active site of the Polβ:oxoA:dGTP complex. 8-kDa domain, fingers domain, palm domain, and thumb domain are shown in purple, green, yellow, and blue, respectively. Hydrogen bonds between nucleobases and interactions with the enzyme amino acids are represented with dashed lines.

Similar to 8-oxoG [40], 8-oxoA presents a mixture of *syn* and *anti* conformers within the Pol β active site. When incorporating complementary dTMP, 8-oxoA adopts the *anti* conformation and forms a canonical Watson–Crick pair (Figure 7) [36]. The ternary structure revealed the closed catalytically competent conformation of the enzyme. Like in the Polβ-(*anti*)8-oxoG:dCTP ternary complex (PDB ID: 3RJI), the accommodation of 8-oxoA in the active site and the adoption of the *anti* conformation require minor rearrangement of the 8-oxoA sugar-phosphate backbone in order to avoid steric clash with the O^8^ atom. This structural alteration may result in a slight decrease in the incorporation efficiency of dTMP opposite 8-oxoA. The distinctive feature of the Polβ:8-oxoA:dTTP complex is the altered orientation of Lys280 [36]. This residue appears to be crucial in stabilizing the lesion within the active site through both stacking interactions with the templating base and a hydrogen bond with Asn37.

The structure of the Polβ:8-oxoA:dGTP ternary complex provides insight into the pro-mutagenic nature of this lesion (Figure 7). The formation of the 8-oxoA:dGMP mismatch in the active site of Pol β does not interfere with a closed catalytically competent conformation of the enzyme. This closed state is achieved mainly through the shift of the α-N helix that recognizes the minor groove. A similar mechanism is observed during formation of the 8-oxoG:dATP and G:dATP pairs [40,134].

The formation of a 8-oxoA:dGTP mispair is accompanied by the adoption of the *syn* conformation by 8-oxoA. As in the case of 8-oxoA:dTTP, the reorganization of the sugar-phosphate backbone is also observed. However, the mechanism of the 8-oxoA:dGTP Hoogsteen base pair formation with Watson–Crick-like geometry is not entirely clear. One potential explanation might be the involvement of the enolate intermediate or the enol tautomer of 8-oxoA in the mismatch formation.

This mismatch is stabilized primarily by Arg283- and Asn279-mediated hydrogen bonds in the Pol β active site. The stabilization of 8-oxoA:dGTP also depends on Lys280-mediated interactions. However, in contrast to the Pol β:8-oxoA:dTTP structure, the shift of the Lys280 side chain observed in the Pol β:8-oxoA:dGTP ternary complex leads to the abrogation of the hydrogen bond with Asp37 and its formation with the 5′-phosphodiester oxygen of 8-oxoA [36].

**Figure 8:**
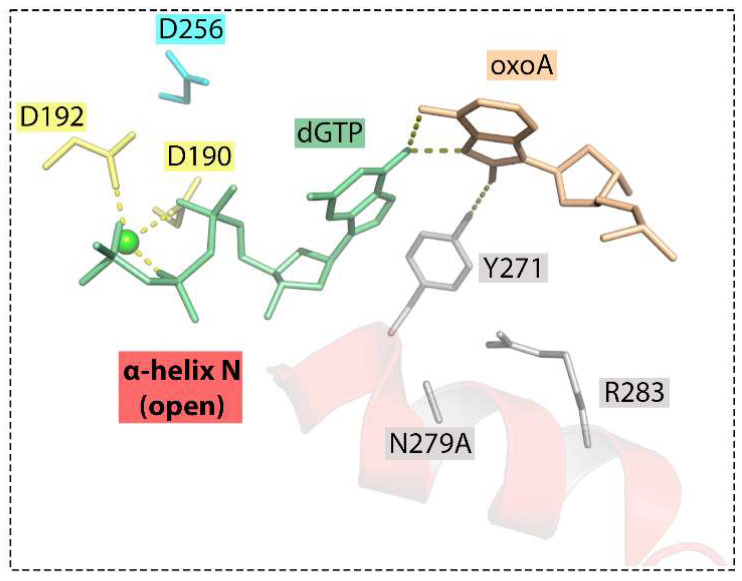
Active site of the Pol β Asn279Ala mutant containing the oxoA:dGTP nascent base pair (PDB ID: 6PKZ) [5]. Interbase bifurcated hydrogen bonds between *syn* 8-oxoA and *syn* dGTP were observed, and the nascent mispair is distorted. The A-site metal ion is absent, and the B-site metal is in a non-optimal position. In the absence of Asn279-mediated contacts, Tyr271 forms a hydrogen bond with O^8^ of *syn* 8-oxoA and stabilizes the mismatch in the active site. Hydrogen bonds between nucleobases or interactions with the enzyme amino acids are represented with dashed lines.

The importance of Asn279 is consistent with enzymatic studies of the Pol β mutant variant with an N279A substitution (Figure 8) [5]. Lack of Asn279, which contacts the minor groove edge of the incoming dNTP, leads to a drastic decrease in the insertion efficiency of both complementary dTMP (through the increase in *K*_m_) and especially non-complementary dGMP opposite 8-oxoA but not opposite the undamaged A. Without Asn279, the α-N helix fails to shift to the nascent pair, and the polymerase remains in the open state. The accommodation of 8-oxoA:dGMP in the active site of mutant Pol β is accompanied by the adoption of the *syn* conformation by both bases of the nascent pair [5].

### 6.2. 8-oxoA Bypass by DNA Polymerase η

All eukaryotic members of the Y family of DNA polymerases—Pol η, Pol κ, Pol ι and REV1—are involved in DNA translesion synthesis. The exceptional ability of Pol η to faithfully replicate thymine–thymine (TT) cyclobutane pyrimidine dimers (CPD) and its key role in protection from UV-induced DNA damage and skin cancer have been well established [135,136,137,138]. Both yeast and human Pol η also efficiently bypasses 8-oxoG in vitro (reviewed in [51]). In yeast, *Ogg1* knockout-dependent mutagenesis is limited by Pol η [139,140]. Pol η also has been suggested to be involved in 8-oxoG-induced mutagenesis suppression in human cells and appears to operate in the same TLS pathway with the B family Pol ζ [141,142,143].

The yeast enzyme has a slight preference for the insertion of the complementary dCMP opposite 8-oxoG in vitro [144,145,146]. A structural study of the yeast Pol η active site revealed that its cleft allows the template lesion to be accommodated in the *anti* conformation, almost without distortion of the polymerase and the bound DNA [147]. Human Pol η carries out a rather mutagenic bypass of the lesion, inserting both dCMP and dAMP, though several studies emphasized that the fidelity of the yeast enzyme is modestly higher compared to the human one [148]. Stabilization of the 8-oxoG:dCMPNPP Watson–Crick base pair in the active site of human Pol η is primarily dependent on the Arg61 residue, which stacks with the base of the incoming nucleotide, as well as on Gln38 [39]. Stabilization of the *syn* orientation of 8-oxoG required to form Hoogsteen base pairs with incoming dATP and dGTP is accomplished through hydrogen bonding between O^8^ and Gln38. In agreement with biochemical studies, there is structural evidence that human Pol η efficiently extends both complementary and non-complementary base pairs [39].

**Figure 9:**
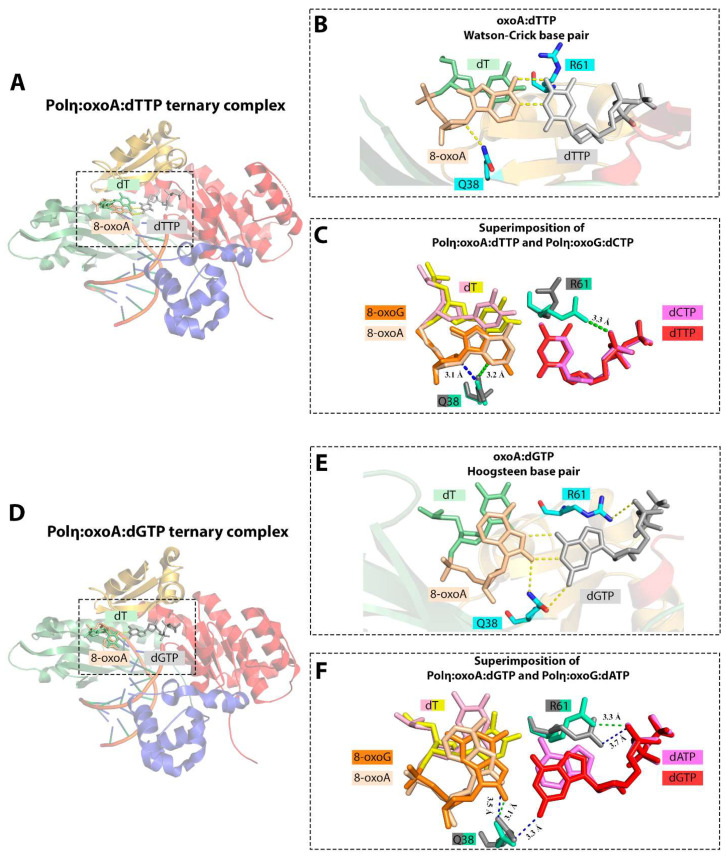
(**A**) Structure of the Polη:oxoA:dTTP ternary complex (PDB ID: 6PL8) [5]. (**B**) Close-up view of the active site of the Polη:oxoA:dTTP complex. (**C**) Conformational differences between 8-oxoA (PDB ID: 6PL8) and 8-oxoG (PDB ID: 4O3P) in the active site of Pol η upon binding complementary incoming nucleotide [5,39]. Superimposition of Polη:oxoA:dTTP and Polη:oxoG:dCTP. Gln38 forms hydrogen bond with the O4 atom of 8-oxoA sugar, whereas in the Polη:oxoG:dCTP complex it is hydrogen bonded to the N3 of 8-oxoG base. (**D**) Structure of the Polη:oxoA:dGTP ternary complex (PDB ID: 6PLC) [5]. (**E**) Close-up view of the active site of the Polη:oxoA:dGTP complex. (**F**) Conformational differences between 8-oxoA (PDB ID: 6PLC) and 8-oxoG (PDB ID: 4O3O) in the active site of Pol η upon binding non-complementary incoming nucleotide [5,39]. Superimposition of Polη:oxoA:dGTP and Polη:oxoG:dATP. Gln38-mediated minor groove interactions with the oxoA:dGTP nascent base pair affect the pro-mutagenic bypass of the oxidized adenine. Palm, fingers, thumb, and little finger domains are shown in red, yellow, blue, and green, respectively. Hydrogen bonds between nucleobases and interactions with the enzyme amino acids are represented with dashed lines.

The catalytic activity of human Pol η is slightly inhibited opposite 8-oxoA; incorporation of dTMP opposite oxoA is ~2.5-fold less efficient than opposite A [36]. Pol η promotes error-prone replication of 8-oxoA incorporating the complementary dTMP only 2-fold more efficiently than the non-complementary dGMP [36]. In addition, Pol η is able to extend from the 8-oxoA:dGMP mismatch without any significant reduction in the catalytic specificity compared to the extension from the 8-oxoA:dTMP base pair [36]. In agreement with the biochemical data, the structural analysis revealed that the Watson–Crick geometry of the 8-oxoA:dTTP pair is well-tolerated by the Pol η active site with no substantial distortion (Figure 9). The only slight differences were found in the minor shift of the 5′-phosphodiester bond of oxoA in order to avoid a steric clash between the 8-oxo moiety and the template nucleotide in the N + 1 position [5]. The conformation of the active site in the Pol η:oxoA:dTTP ternary complex strongly resembles the Pol η:G:dCTP complex. Like in Pol β, 8-oxoA in the Pol η active site adopts the *anti* conformation and forms the Watson–Crick base pair with the incoming dTTP. Stacking interactions between the nascent base pair and downstream templating nucleobase (T) and Gln38-mediated hydrogen bonding stabilize the oxoA:dTTP pair [5].

8-oxoA adopts the *syn* conformation and forms non-canonical Hoogsteen base pair with an incoming dGTP (Figure 9). The Gln38-mediated minor groove hydrogen bonds also play a key role in the formation and stabilization of oxoA:dGTP base pair with a wobble configuration [5]. Unlike in the Pol η:oxoG:dATP ternary complex, Gln38-mediated interactions in Pol η:oxoA:dGTP complex appear not to be limited to the template lesion, and Gln38 also makes contacts with the incoming dGTP. The data are consistent with the results of the Pol η mutant variant analysis, demonstrating that the substitution Q38A leads to only a 4-fold reduction in dAMP incorporation opposite 8-oxoG but to a 55-fold reduction in dGMP incorporation opposite oxoA [5].

The active site residue Arg61 plays an equivocal role. In the complex with T:dGTP, major groove interactions mediated by Arg61 force this residue to adopt a conformation unfavorable for catalysis, thereby facilitating the discrimination against this mismatch in the Pol η active site. However, in the complex with 8-oxoA:dGTP, the conformation of Arg61, interacting with the phosphate oxygen of the incoming triphosphate, does not prevent a relatively efficient insertion of non-complementary dGMP opposite the template lesion.

### 6.3. 8-oxoA Bypass by Dpo4

The archaeal *Sulfolobus solfataricus* Dpo4, structurally reminiscent of Pol η and Pol κ, is commonly employed as a model for studying translesion DNA synthesis. To date, this is the third DNA polymerase whose structure in a complex with 8-oxoA-containing DNA has been solved [46,47]. It was demonstrated that the incorporation of the complementary dTMP opposite 8-oxoA is guided by the Arg331- and Arg332-mediated hydrogen contacts stabilizing 8-oxoA in the *anti* conformation. However, formation of a canonical Watson–Crick base pair apparently requires a conformational alteration of DNA downstream of the templating lesion (in the N + 1 and N + 2 positions), as in the case of 8-oxoG lesion [46]. The highly efficient elongation from the 8-oxoA:dTMP pair in the biochemical assay is consistent with the obtained structural findings, demonstrating that accommodation of the pair following damage occurs without an apparent conformational distortion [47].

The solved Dpo4:8-oxoA:dGTP complex provides some insight into the pro-mutagenic nature of 8-oxoA. The active site accommodates the 8-oxoA:dGTP Hoogsteen base pair, apparently with three hydrogen interbase bonds, suggesting that one of the bases is in a minor tautomeric form [46]. The hydrogen bond between Arg332 and the 8-oxo group of 8-oxoA was abrogated in this ternary complex. It has also been noted that the bulk of two purine rings may interfere with the correct positioning of the 3′-OH terminus of the primer and its coordination with Mg^2+^ in the A-site [46]. According to the kinetic analysis, this mismatch is extended 5-fold less efficiently compared to the 8-oxoA:dTMP base pair.

Significant differences were uncovered by comparing insertion and extension structures. Thus, in the latter, template lesion adopted the *anti* conformation leading to rearrangement of the hydrogen interbase bonding. It was also unexpected that the primer terminus, in order to avoid a steric clash between *anti* 8-oxoA and dGTP, shifts to an extrahelical site where the flipped-out conformation of dG is stabilized by a network of minor groove hydrogen bonding and Glu106- and Tyr108-mediated contacts [47].

The above-discussed BER and TLS directly involved in genome protection are likely not the only mechanisms neutralizing the harmful effects of oxidized adenine. Depletion of glutathione causes significant 8-oxoG and 8-oxoA accumulation in vivo [149]. Therefore, an antioxidant vitagene network may also control the harmful effects of oxidative stress.

## 7. Unresolved Questions

Oxidative DNA damage is a well-known source of mutagenesis. Recent studies demonstrated that 8-oxoA is a stable and abundant lesion induced by oxidative stress. Given the frequency of 8-oxoA in the genome, dual-coding properties and other deleterious effects of its persistence in the genome, the accumulation of 8-oxoA, along with 8-oxoG, may pose a significant threat to genomic stability. Although genotoxicity studies have demonstrated mutagenic potential of 8-oxoA in eukaryotic cells that is potentially of clinical relevance, very little biochemical and bioinformatics data about the mechanism of 8-oxoA-induced mutagenesis are available. Proposed association of SBS17a and SBS17b cancer signatures with 8-oxoA-specific mutagenesis requires bioinformatic verification. DNA polymerases responsible for the error-prone bypass of 8-oxoA in DNA and incorporation of 8-oxodAMP from the nucleotide pool are yet to be identified. Since the lagging-strand enrichment for the SBS17a and SBS17b signatures has been demonstrated, Pol δ is a possible candidate enzyme for biochemical studies.

Another possible source of 8-oxoA-induced mutagenesis is repair and translesion DNA polymerases. For example, Y-family Pol η and X-family Pol were shown to efficiently incorporate dGMP opposite 8-oxoA in vitro [36]. Interestingly, the accuracy of 8-oxoG bypass by repair and TLS enzymes is significantly enhanced in the presence of auxiliary proteins such as PCNA, RFC, and RPA in vitro [150]. The 8-oxoG-induced mutagenesis assay in yeast also supported the significance of Pol η interaction with PCNA [151]. TLS opposite 8-oxoA can be affected by Me^2+^ ions. Some DNA polymerases (Pol ι, Pol λ, primase-polymerase PrimPol) efficiently utilize Mn^2+^ ions [152,153,154,155,156,157] which often increases the efficiency but decreases the accuracy of TLS. Mn^2+^ ions induce Watson–Crick-like G:T mismatch in Pol β (with the pH-dependent insertion efficiency) suggesting the base pairing is ionization-mediated; however, the subsequent slow protonation of the ionized form resulting in guanine enolization is not excluded [158]. Moreover, DNA sequence context significantly affects the accuracy of nucleotide incorporation opposite 8-oxoG by human DNA polymerases [159]. The emerging evidence suggests that DNA sequence context is also a key factor modulating the fidelity of TLS opposite 8-oxoA in mammalian cells. Therefore, the role of accessory proteins, Me^2+^ ions, and DNA sequence context should be taken into consideration in future biochemical studies.

Remarkably, 8-oxoA is not mutagenic in some studies but exhibits mutagenic properties comparable to 8-oxoG in others. It is well-known that the accuracy of nucleotide incorporation by many DNA polymerases depends on DNA sequence-context [160,161] and DNA polymerases may have specific “mutable motifs” demonstrating “mutation signatures” [162,163]. It is quite possible that 8-oxoA-assosiated mutagenesis is sequence-dependent and limited to specific mutagenesis sites or hotspots, including cancer driver genes. However, a possible mechanism of sequence-dependent mutagenesis opposite 8-oxoA is unclear. Certain nucleotide combinations may have a universal effect on the site-specific mutation rate [161,164,165]. In particular, neighbor base stacking interactions and the relative G:C proportions can increase local thermodynamic stability causing stabilization of nucleotide mispairing during DNA synthesis and interfering with nucleotide proofreading of mispaired bases [165]. Strikingly, signatures SBS17a and SBS17b are prone to form hotspots 10–70 times more often than the other cancer signatures. This high propensity to form hotspots remains unexplained but was suggested to be associated with some small local genomic features [166]. Prokaryotic and eukaryotic aerobic organisms have a variety of versatile mechanisms to alleviate the effects of a plethora of DNA damaging agents. Among them, BER is likely the main pathway preventing the harmful effects of 8-oxoA. However, the exact repair pathways are yet to be determined. To date, a limited spectrum of human DNA-glycosylases capable of excising 8-oxoA in vitro has been identified (TDG, OGG1, NEIL1), but the biological significance of these findings remains uncertain. Studies with reporter genes in living cells may shed light on 8-oxoA mutagenesis spectra and repair mechanisms operating on this lesion.

## Figures and Tables

**Table 1:** Accuracy of 8-oxoA bypass in prokaryotes.

In Vitro/ In Vivo	Enzyme/ Organism	Sequence	Assay	Accuracy	Ref.
in vitro	KF	3′-…TCGGXTGGT…-5′	Primer extension	Effectively bypasses	[30]
		5′-CCTTCXCTAC…-3′ 5′-GTTGXGTAC…-3′	Primer extension and steady-state kinetics	Preferentially inserts dTMP and small amounts of dAMP and dGMP; F_ins_ dTTP~500–1000x > F_ins_ dAMP/dGMP; F_ext_ oxoA:T~630–3000x > F_ext_ 8-oxoA:A and 8-oxoA:C	[43]
		5′-…GGCC**XAG**-3′ (*HRAS* template)	Primer extension	Inserts only dTMP	[42]
		5′-…GGTCXTCGG-3′	Primer extension	Preferentially inserts dTMP and small amounts of dAMP	[44]
	Taq pol	3′-…TCGGXTGGT…-5′	Primer extension	Inserts only dTMP	[30]
		5′-…GGCC**XAG**-3′ (*HRAS* template)	Primer extension	Inserts only dTMP	[42]
	Dpo4	5′-TCATXGAAT…-3′ 5′-TTCATXGAAT…-3′	Steady-state kinetics	F_ins_ dTMP~14x > F_ins_ dGMP F_ins_ 8-oxoA:dGMP~320x > F_ins_ A:dGMP F_ext_ 8-oxoA:T~5x > F_ext_ 8-oxoA:G	[46,47]
in vivo	*E. coli*	5′-GCTXG-3′	Mutagenesis assay	MF~0.2–0.3%	[41]

X = 8-oxoA, the *HRAS* mutational hot spot sequence is shown in bold. Mutation frequency (MF) = [number of analyzed mutant colonies/total analyzed colonies] × 100%. Frequency of insertion (F_ins_) and frequency of extension (F_ext_) = (*k*_cat_/*K*_m_) [mismatch]/(*k*_cat_/*K*_m_) [correct pair] or (V_max_/*K*_m_) [mismatch]/(V_max_/*K*_m_) [correct pair].

**Table 2:** Accuracy of 8-oxoA bypass in mammalian cells.

In Vitro/ In Vivo	Enzyme/ Cell Line	Sequence	Assay	Accuracy	Ref.
In vitro	Pol α	5′-CCTTCXCTAC…-3′ 5′-GTTGXGTAC…-3′	Primer extension and steady-state kinetics	Preferentially inserts dTMP and small amounts of dGMP; F_ins_ dTTP~10x > dGTP; F_ext_ 8-oxoA:T and 8-oxoA:G~280–3500x < than for KF	[43]
		5′-…GGCC**XAG**-3′ (*HRAS* template)	Primer extension	dTMP > dGMP *	[42]
	Pol β	5′-CCTTCXCTAC…-3′ 5′-GTTGXGTAC…-3′	Primer extension and steady-state kinetics	Preferentially inserts dTMP and small amounts of dGMP; F_ins_ dTTP~18x > dGTP	[43]
		5′-…GGCC**XAG**-3′ (*HRAS* template)	Primer extension	dTMP > dGMP > dAMP *	[42]
		5′-…TACGXCGCA…-3′	Steady-state kinetics	F_ins_ 8-oxoA:dTMP ~2.5x < F_ins_ A:dTMP k_cat_/K_m_ 8-oxoA:T~4x > k_cat_/K_m_ 8-oxoA:G	[36]
	Pol η	5′-…TACGXCGCA…-3′	Steady-state kinetics	F_ins_ dTTP~2x > dGTP; k_cat_/K_m_ 8-oxoA:T~1.1x > k_cat_/K_m_ 8-oxoA:G	[36]
	HeLa and COS-7 cell extracts	5′-CCTTCXCTAC…-3′	Primer extension	Inserts only dTMP	[48]
In vivo	NIH 3T3 cells	5′-…GGCC**XAG**-3′ (*HRAS* template)	Mutagenesis assay	A → C in 55% clones, A → G in 10% clones	[42]
	COS-7 cells	5′-…TC**CTX**GCCT…-3′ (non-coding strand of *HRAS* template)	Mutagenesis assay	MF~1.2%	[50]
		5′-…CCTGXCCTC…-3′		MF~0.24%	

X = 8-oxoA, the *HRAS* mutational hot spot sequence is shown in bold. Mutation frequency (MF) = [number of analyzed mutant colonies/total analyzed colonies] × 100%. Frequency of insertion (F_ins_) and frequency of extension (F_ext_) = (*k*_cat_/*K*_m_) [mismatch]/(*k*_cat_/*K*_m_) [correct pair] or (V_max_/*K*_m_) [mismatch]/(V_max_/*K*_m_) [correct pair]. *—no quantitative data.

## Data Availability

Not applicable.
